# Liver Cirrhosis and Diabetes Mellitus Are Risk Factors for *Staphylococcus aureus* Infection in Patients with Healthcare-Associated or Hospital-Acquired Pneumonia

**DOI:** 10.1155/2016/4706150

**Published:** 2016-02-22

**Authors:** Huang-Pin Wu, Chien-Ming Chu, Chun-Yao Lin, Chung-Chieh Yu, Chung-Ching Hua, Teng-Jen Yu, Yu-Chih Liu

**Affiliations:** ^1^Division of Pulmonary, Critical Care and Sleep Medicine, Chang Gung Memorial Hospital, 222 Maijin Road, Anle District, Keelung 20401, Taiwan; ^2^Chang Gung University College of Medicine, Taoyuan, Taiwan

## Abstract

*Background.* The risk factors for* Staphylococcus aureus* (*S. aureus*) pneumonia are not fully identified. The aim of this work was to find out the clinical characteristics associated with* S. aureus* infection in patients with healthcare-associated pneumonia (HCAP) and hospital-acquired pneumonia (HAP), which may be applicable for more appropriate selection of empiric antibiotic therapy.* Methods.* From July 2007 to June 2010, patients who were admitted to the intensive care unit with severe HCAP/HAP and severe sepsis were enrolled in this study. Lower respiratory tract sample was semiquantitatively cultured. Initial broad-spectrum antibiotics were chosen by Taiwan or American guidelines for pneumonia management. Standard bundle therapies were provided to all patients according to the guidelines of the Surviving Sepsis Campaign.* Results.* The most frequently isolated pathogens were* Pseudomonas aeruginosa*,* S. aureus*,* Acinetobacter baumannii*,* Klebsiella pneumoniae*, and* Escherichia coli*. Patients with positive isolation of* S. aureus* in culture had significantly higher history of liver cirrhosis and diabetes mellitus, with odds ratios of 3.098 and 1.899, respectively. The* S. aureus* pneumonia was not correlated with history of chronic obstructive pulmonary disease, hypertension, and hemodialysis.* Conclusion.* Liver cirrhosis and diabetes mellitus may be risk factors for* S. aureus* infection in patients with severe HCAP or HAP.

## 1. Introduction

In present medical practice, severe pneumonia with CURB-65 score (confusion, uremia, respiratory rate, low blood pressure, and age 65 years or greater) of ≧ 3 points comprised more than 16% and 25% of 30-day mortality rate among community-acquired pneumonia (CAP) and healthcare-associated pneumonia (HCAP), respectively [1]. Initial broad-spectrum empiric antibiotic selection is important because inadequate or delayed antibiotic treatment results in high mortality. However, overuse of empirical broad-spectrum antibiotics can create multidrug-resistant (MDR) pathogens and contribute to antibiotic-induced complications.

Selection of antibiotics for initial empirical therapy is based on prediction of the most likely pathogens and knowledge of local susceptibility. Guidelines provide some risk factors for specific pathogens [2, 3]. This can help us decide initial empiric antibiotics. In general, physicians prescribe initial antibiotics which should be effective for* Pseudomonas aeruginosa *(*P. aeruginosa*) and MDR pathogens in patients combining HCAP and hospital-acquired pneumonia (HAP) with severe sepsis. If* Staphylococcus aureus *(*S. aureus*) is considered, vancomycin, linezolid, or teicoplanin is added due to high risk of methicillin-resistant* S. aureus* (MRSA). The Infectious Diseases Society of America and American Thoracic Society suggest risk factors of* S. aureus* pneumonia in patients with hemodialysis, lung abscess, structure lung disease, injection drug use, prior influenza, prior antibiotic therapy, and endobronchial obstruction [2].

However, the best indicator of* S. aureus* infection is that Gram staining shows a single/predominant Gram-positive coccus with clustered and polymorphonuclear cells. Then,* S. aureus* infection could be confirmed by following culture report. But culture report needs 3-4 days. Patients with late-onset HAP and HCAP are at greater risk for infection with MDR pathogens [3]. Thus, it becomes an important issue whether critically ill patients with severe HAP and HCAP have probability of* S. aureus* infection in present clinical practice.

We designed a prospective observational study to determine the prevalence and epidemiologic risk factor of* S. aureus* infection among patients admitted to intensive care unit (ICU) due to severe HCAP and HAP.

## 2. Materials and Methods

### 2.1. Subjects

From July 2007 to June 2010, patients who were admitted to the ICU at Chang Gung Memorial Hospital, Keelung, due to HCAP and HAP with severe sepsis or septic shock were enrolled in this study. The ICU is a medical and closed unit in our hospital. This study was approved by the Institutional Review Board at Chang Gung Memorial Hospital (96-0132B, 97-0121C, and 98-1682C). The following patient data were recorded within the first 3 days after admission: age; gender; medical history; lower respiratory tract sample for semiquantitative culture; Acute Physiology and Chronic Health Evaluation (APACHE) II score; and adverse events. History of intravenous drug use and prior antibiotic use within 30 days was recorded according to the statement of patient or patient's family. Endotracheal aspirates were used as lower respiratory tract samples for culture initially. If cultures were negative, bronchoalveolar lavages were performed to detect pathogens. Samples contaminated by upper airway secretions, as reflected by more than 10 squamous epithelial cells/low power field, were excluded.

### 2.2. Disease Definitions

Pneumonia was defined as new abnormal infiltration on chest radiograph with respiratory symptoms or fever. Pneumonia was classified as HCAP and HAP according to guidelines [3]. HCAP includes any patient who was hospitalized in an acute care hospital for two or more days within 90 days of the infection; resided in a nursing home or long-term care facility; received recent intravenous antibiotic therapy, chemotherapy, or wound care within the past 30 days of the current infection; or attended a hospital or hemodialysis clinic. HAP is defined as pneumonia that occurs 48 hours or more after admission, which was not incubating at the time of admission. Severe sepsis and septic shock were defined according to the criteria established in the Consensus Conference [4]. Systemic inflammatory response syndrome (SIRS) was defined as fulfillment of two or more of the following criteria: (1) body temperature >38°C or <36°C; (2) respiratory rate >24 breaths/minute; (3) heart rate >90 beats/minute; and (4) white blood count >12,000/*μ*L or <4000/*μ*L or >10% bands. Sepsis was defined as SIRS according to a confirmed or suspected microbial etiology. Severe sepsis was defined as sepsis with one or more dysfunctional organs or hypotension. Septic shock was defined as sepsis with hypotension unresponsive to fluid resuscitation, which further required vasopressors to maintain blood pressure during the first three days following ICU admission. Acute renal failure was diagnosed as a rapidly rising serum creatine level ≥0.5 mg/dL over the baseline value [5]. Disease severity was assessed with the APACHE II score [6]. Survivors were defined as patients who were alive 30 days after ICU admission.

### 2.3. Treatment

Standard bundle therapies, including fluid resuscitation, broad-spectrum antibiotics, drainage, blood transfusion, sedation/paralysis, blood glucose control, hemodialysis, stress ulcer prophylaxis, and basic support, were provided to all patients according to the recommended guidelines [7]. Initial broad-spectrum antibiotics were chosen by the Taiwan Guidelines for Pneumonia Management (2007 version) or Guidelines of American Thoracic Society [3].

### 2.4. Statistical Analysis

Statistical analysis was performed with the Statistical Package for the Social Sciences (SPSS) V17.0 for Windows (SPSS, Inc., Illinois, USA). Differences for the continuous variables between the two groups were analyzed by Mann-Whitney test. Differences for categorical variables between the two groups were compared by Pearson chi-square test or Fisher's exact test. General linear model was used to determine the associations of* S. aureus* pneumonia among all factors. A *p* value of less than 0.05 was considered statistically significant. Odds ratio is the odds that a patient is exposed to the risk factor divided by the odds that a control is exposed to.

## 3. Results

**Figure 1:**
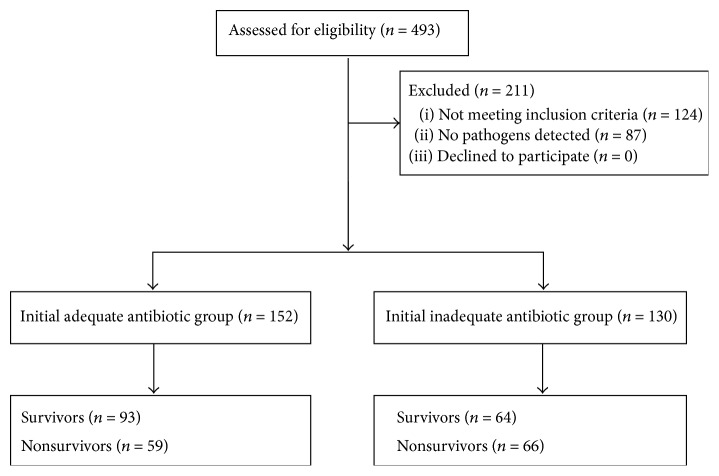
During the study period, 493 patients with severe sepsis and septic shock were screened, and 211 patients were excluded. The reasons for exclusion included nonpneumonia infection and no detectable pathogens in lower respiratory tract sample for culture. A total of 282 patients were enrolled for analysis. No patients withdrew. Initially, 152 and 130 patients received adequate and inadequate antibiotic therapy, respectively. Fifty-nine and 66 patients died in adequate and inadequate antibiotic groups, respectively.

During the study period, 493 patients with severe sepsis or septic shock were screened (Figure 1). A total of 282 patients were enrolled for analysis, and 211 patients were excluded. The reasons for exclusion included nonpneumonia infection (*n* = 73), coisolated pathogens (*n* = 51), and no detectable pathogens (*n* = 87) in lower respiratory tract sample for culture. Patients with coisolated pathogens and no detectable pathogens were excluded to reduce the possible bias maximally due to concerns of pathogen colonization and unknown pathogens. No patients withdrew. Initially, 152 and 130 patients received adequate and inadequate antibiotic therapy, respectively. Fifty-nine and 66 patients died in adequate and inadequate antibiotic groups, respectively. Clinical characteristics of HCAP or HAP patients with severe sepsis are shown in Table 1. Patients with* S. aureus* pneumonia had higher percentage of liver cirrhosis and diabetes mellitus history. Percentages of history of chronic obstructive pulmonary disease (COPD), congestive heart failure (CHF), hypertension, and hemodialysis were similar between presence and absence of* S. aureus* pneumonia. There were no differences in age, APACHE II score, sex, adverse events, and 30-day mortality rates between patients with and without* S. aureus* infection.

Table 2 shows the isolated pathogens with initial adequate or inadequate antibiotic treatment. The most frequently isolated pathogens, in decreasing order, were* P. aeruginosa*,* S. aureus*,* Acinetobacter baumannii*,* Klebsiella pneumoniae *(*K. pneumoniae*),* Escherichia coli* and so forth. All patients with* Streptococcus pneumonia* (*S. pneumonia*) infection were prescribed adequate antibiotic treatment initially. Only* S. pneumonia* infection was associated with 30-day mortality (Table 3). Patients with* S. pneumonia* infection had higher survivor rate. There was no difference in mortality between other pathogens and mortality.

After regression analysis, patient's history of CHF, liver cirrhosis, and diabetes mellitus were independent factors associated with* S. aureus* pneumonia (Table 4). The odds ratios of CHF, liver cirrhosis, and diabetes mellitus were 2.242, 3.098, and 1.899, respectively. The* S. aureus* pneumonia was not correlated with history of prior antibiotic use, COPD, hypertension, and chronic renal failure. The* S. aureus* pneumonia was also not associated with age, gender, APACHE II score, adverse events, and mortality.

## 4. Discussion

The present investigation is the first to find that history of CHF, liver cirrhosis, and diabetes mellitus increased the risk of* S. aureus* infection in patients with severe HAP or HCAP. Immunodeficiency in cirrhosis is multifactorial and might not be reversed by interventions. Polymorphonuclear leukocyte dysfunction and complement deficiency are well known as main causes leading to infection predisposition [8].* S. aureus* has been reported to be the most frequently (27.4%) isolated pathogen from blood and ascites fluid in patients with liver cirrhosis [9]. From the database of a surveillance study of* S. aureus* infections, bacteremia and bone infection were more frequent in the chronic liver disease group when compared with the other-disease group [10]. After multivariate analysis, chronic liver disease was a factor significantly associated with 30-day mortality. However, there was no difference in pneumonia rate between the chronic liver disease and other-disease groups. This was dissimilar from the current study's findings. In this research, we found that HCAP or HAP patients with liver cirrhosis had higher percentage of* S. aureus* infection. More studies may be needed to clarify the association between* S. aureus* pneumonia and liver cirrhosis.

Compared with nondiabetes patients with severe pneumonia, diabetes patients had 1.899 of odds ratio for* S. aureus* infection in this study. Hyperglycemia promoted respiratory* S. aureus* infection, and metformin modified glucose flux across the airway epithelium to limit hyperglycemia-induced bacterial growth [11]. Shorr et al. developed a risk score to identify* S. aureus* in patients with HCAP [12]. Female with diabetes was one of eight variables in the final risk score. Furthermore, diabetes could be thought of as immunosuppressive disease, a risk factor of methicillin-resistant* S. aureus* infection (MRSA) [13]. However, in a study of healthcare-associated infection, Erben et al. found that diabetes mellitus was not a risk factor for* S. aureus* pneumonia [14]. Diabetes mellitus was a risk factor for* S. aureus* infection at surgical site. More studies may be necessary to determine the relationship between* S. aureus* pneumonia and diabetes mellitus.

In this work,* S. aureus *infection was the second most frequently found pathogen in patients with severe pneumonia. In Korea and Japan, the frequent pathogens in HCAP, in decreased order, were* S. pneumonia*,* S. aureus*,* P. aeruginosa*, and* K. pneumoniae* [15, 16]. Also,* S. pneumonia*,* P. aeruginosa*, and* S. aureus* were the frequently detected pathogens in HCAP in Spanish [17].

In this study, patients with chronic obstructive pulmonary disease (COPD) did not have higher risk of* S. aureus* infection or colonization. That is similar to Infectious Diseases Society of America/American Thoracic Society Consensus Guidelines on the Management of Community-Acquired Pneumonia in Adults [2]. However, in a retrospective chart review study, the risk for MRSA was increased by tobacco use (OR = 2.31, CI 1.23–4.31) and COPD (OR = 3.76, CI 1.74–8.08) in CAP patients [18], and the risk of COPD disappeared in HAP patients. Thus, whether COPD increases risk for* S. aureus* in HCAP is still unclear. About the history of CHF, we did not find published reports or studies showing the presence of correlation between CHF and* S. aureus*. Hemodialysis was not associated with* S. aureus* pneumonia in this work. This may be due to the fact that more than 90% identified* S. aureus* events were bloodstream infection [19]. Although hemodialysis did not significantly increase the risk of* S. aureus* pneumonia, patients with end-stage renal disease and hemodialysis patients had increased risk of* S. aureus* bacteremia [20].

As we know, inadequate empiric antimicrobial treatment may be associated with excess hospital mortality in pneumonia [3]. However, this point might not be constant after bundle therapy of severe sepsis. In our previous study, there was no difference in mortality between initial adequate and inadequate antibiotic therapy in patients with severe sepsis [21]. In this work, even though most of patients were prescribed initial adequate antibiotic therapy for* P. aeruginosa* and* K. pneumoniae*, their mortality rates did not decrease significantly. Only patients with* S. pneumonia* infection had significantly higher survival rate. The most likely reason might be that all patients with* S. pneumonia* infection were prescribed adequate empiric antibiotic therapy.

There are two limitations in this study. First, the accuracy of diagnosing pathogens is based on lower respiratory tract quantitative cultures of endotracheal aspirates, protected specimen brush, or bronchoalveolar lavage samples. The lower respiratory tract culture in our hospital is semiquantitative. Further, a positive culture cannot always distinguish a pathogen from a colonizing organism [3]. The culture results in this work may include colonizing pathogens. Second, there is a possible bias in statistical analysis. The percentage of history of CHF was low in this study. That made the statistical power low in regression analysis.

In conclusion, the traditional risk factors for* S. aureus* infection may change or vary. This study concludes that liver cirrhosis and diabetes mellitus may be risk factors for* S. aureus* infection in patients with HCAP or HAP. Using these features may allow us to select adequate empiric antibiotics.

## Figures and Tables

**Table 1:** Clinical characteristics between presence and absence of *S. aureus* infection in HCAP or HAP patients with severe sepsis.

	*S. aureus* (*N* = 63)	No *S. aureus* (*N* = 219)	*p* value
Age, years^*∗*^	73.7 ± 14.4	73.9 ± 13.0	0.938
APACHE II score^*∗*^	26.0 ± 6.7	27.0 ± 7.8	0.477
Sex, number (%)			0.916
Male	43 (68.3)	151 (68.9)	
Female	20 (31.7)	68 (31.1)	
History, number (%)			
Prior antibiotic use	20 (31.7)	64 (29.2)	0.700
Intravenous drug use	0 (0.0)	0 (0.0)	†
COPD	9 (14.3)	47 (21.5)	0.208
CHF	10 (15.9)	17 (7.8)	0.054
Hypertension	26 (41.3)	90 (41.1)	0.980
Liver cirrhosis	11 (17.5)	14 (6.4)	0.006
Hemodialysis	4 (6.3)	20 (9.1)	0.485
Diabetes mellitus	27 (42.9)	62 (28.3)	0.029
Adverse events, number (%)			
RF with intubation and MV	63 (100.0)	219 (100.0)	†
GI bleeding	8 (12.7)	33 (15.1)	0.638
Shock	31 (49.2)	100 (45.7)	0.619
New arrhythmia	4 (6.3)	13 (5.9)	1.000
Acute renal failure	27 (42.9)	92 (42.0)	0.904
Jaundice	7 (11.1)	17 (7.8)	0.401
Thrombocytopenia	23 (36.5)	83 (37.9)	0.841
30-day mortality, number (%)	25 (39.7)	100 (45.7)	0.400

*S. aureus*: *Staphylococcus aureus*; HCAP: healthcare-associated pneumonia; HAP: hospital-acquired pneumonia; APACHE: Acute Physiology and Chronic Health Evaluation; COPD: chronic obstructive pulmonary disease; CHF: congestive heart failure; RF: respiratory failure; MV: mechanic ventilator; and GI: gastrointestinal

^*∗*^Data is shown as mean ± standard deviation.

^†^No statistic was computed because variable is a constant.

**Table 2:** Pathogens in severe pneumonia between adequate and inadequate antibiotic use.

Pathogens	Adequate *N* (% of total)	Inadequate *N* (% of total)	Total *N* (% of pathogens)
*Pseudomonas aeruginosa*	53 (74.6)	18 (25.4)	71 (25.1)
*Staphylococcus aureus*	31 (49.2)	32 (50.8)	63 (22.3)
MSSA	10 (15.9)	3 (4.8)	13 (4.6)
MRSA	21 (33.3)	29 (46.0)	50 (17.7)
*Acinetobacter baumannii*	18 (35.3)	33 (64.7)	51 (18.0)
*Klebsiella pneumoniae*	32 (72.7)	12 (27.3)	44 (15.5)
*Escherichia coli*	21 (67.7)	10 (32.3)	31 (11.0)
*Streptococcus pneumonia*	11 (100.0)	0 (0.0)	11 (3.8)
*Stenotrophomonas maltophilia*	3 (42.9)	4 (57.1)	7 (2.5)
*Enterobacter*	3 (60.0)	2 (40.0)	5 (1.8)

MSSA: Methicillin-Sensitive *Staphylococcus aureus*; MRSA: Methicillin-Resistant *Staphylococcus aureus*.

**Table 3:** Difference in mortality among pathogens in severe pneumonia.

Pathogens	Survivors	Nonsurvivors	*p* value
*Pseudomonas aeruginosa*			0.338
Yes	34	28	
No	114	97	
*Staphylococcus aureus*			0.400
Yes	38	25	
No	119	100	
*Acinetobacter baumannii*			0.617
Yes	30	21	
No	127	104	
*Klebsiella pneumoniae*			0.868
Yes	25	19	
No	132	106	
*Escherichia coli*			0.776
Yes	18	13	
No	139	112	
*Streptococcus pneumonia*			0.026
Yes	10	1	
No	147	124	
*Stenotrophomonas maltophilia*			0.137
Yes	6	1	
No	151	124	
*Enterobacter*			0.658
Yes	2	3	
No	155	122	

**Table 4:** General linear model to predict *Staphylococcus aureus* pneumonia.

Variables	*B*	*p* value	95% confidence interval	
			Lower	Upper
Age, years	0.000	0.839	−0.004	0.004
APACHE II score	−0.005	0.218	−0.012	0.003
Sex, male	−0.014	0.810	−0.124	0.097
History				
Prior antibiotic use	−0.010	0.859	−0.119	0.099
COPD	−0.053	0.434	−0.185	0.080
CHF	0.175	0.043	0.006	0.345
Hypertension	−0.026	0.639	−0.135	0.083
Liver cirrhosis	0.334	0.003	0.117	0.552
Hemodialysis	−0.080	0.398	−0.267	0.107
Diabetes mellitus	0.113	0.049	0.001	0.225
Adverse events				
GI bleeding	−0.076	0.317	−0.224	0.073
Shock	0.060	0.304	−0.054	0.173
New arrhythmia	0.006	0.957	−0.205	0.216
Acute renal failure	−0.012	0.831	−0.121	0.097
Jaundice	−0.097	0.392	−0.319	0.125
Thrombocytopenia	0.000	0.989	−0.111	0.110
30-day mortality, death	−0.023	0.692	−0.136	0.090
